# Supplementary material for: Naphthoquinones Oxidize H2S to Polysulfides and Thiosulfate, Implications for Therapeutic Applications
Source: Int J Mol Sci. 2022 Oct 31;23(21):13293. doi: 10.3390/ijms232113293 (PMC9657496; doi:10.3390/ijms232113293)

Supplemental Information
Naphthoquinones Oxidize H_2S to Polysulfides and Thiosulfate, Implications for Therapeutic Applications

Kenneth R. Olson[a,b,*] Kasey J. Clear[c], Paul J. Derry[d], Yan Gao[a], Zhilin Ma[a,b], Nathaniel M. Cieplik[a,b], Alyssa Fiume[a,b], Dominic J. Gaziano[a,b], Stephen M. Kasko[a,b], Kathleen Narloch[a,b], Cecilia L. Velander[a,b], Ifeyinwa Nwebube[a,b], Collin J. Pallissery[a,b], Ella Pfaff[a,b], Brian P. Villa[a,b], Thomas A. Kent[d,e,f], Gang Wu[g], Karl D. Straub[h,i]

a. Indiana University School of Medicine - South Bend Center, South Bend, Indiana 46617 USA
b. Department of Biological Sciences, University of Notre Dame, Notre Dame, IN 46556, USA
c. Department of Chemistry and Biochemistry, Indiana University South Bend, South Bend, Indiana 46615 USA
d. Center for Genomics and Precision Medicine, Institute of Biosciences and Technology, Texas A&M Health Science Center, Houston, Texas 77030, USA
e. Department of Chemistry, Rice University, Houston, Texas 77005, United States
f. Stanley H. Appel Department of Neurology, Houston Methodist Hospital and Research Institute, 6560 Fannin Street, Houston, Texas 77030, United States
g. Department of Internal Medicine, University of Texas – McGovern Medical School, Houston, Texas 77030, USA
h. Central Arkansas Veteran's Healthcare System, Little Rock, AR 72205, USA
i. Departments of Medicine and Biochemistry, University of Arkansas for Medical Sciences, Little Rock, AR 72202, USA

Running Head: Sulfur Metabolism by Naphthoquinones

***Address correspondence to:**
Kenneth R. Olson, Ph.D.
Indiana University School of Medicine -South Bend
Raclin Carmichael Hall
1234 Notre Dame Avenue
South Bend, IN 46617
Phone: (574) 631-7560
Fax: (574) 631-7821
e-mail: olson.1@nd.edu

Supplemental Figures

Supplemental Figure S1. Stability and relative distribution of inorganic sulfur compounds derivatized with IAM or TME-IAM after dissolving the respective salt and subjected to LCMS analysis. (**A**) H_2S (300 µM as Na_2S) and H_2S_2 as an impurity in Na_2S, derivatized with IAM in 21% O_2 are relatively stable for 60 min, whereas the area under the curve (AUC) for all polysulfides produced from 100 µM K_2S (K_2S_n) derivatized with TME-IAM in either 21% (**B**) or <1% O_2 (**C**) declines rapidly. (**D-F**) distribution of H_2S and polysulfides (S_n, n=1-6) as a percent of total sulfur from 100 µM K_2S_n derivatized with IAM in 21% O_2 (**D**) or with TME-IAM in 21% O_2 (**E**) or <1% O_2 (**F**). Considerably less H_2S is present in TME-IAM derivatized samples, O_2 has little effect on TME-IAM derivatization.

Supplemental Figure S2. Oxidation of 300 µM to polysulfides (SSP4 fluorescence) by; (**A**) 2-methoxy-1,4-naphthoquinone, (**B**) anthraquinone, (**C**) phylloquinone, (**D**) menaquinone, and (**E**) lawsone. Mean +SE, n=4 wells per treatment. *, p <0.05; ***, p <0.001 compared to SSP4 plus H_2S.

Supplemental Figure S3. 1,4-Naphthoquinone (1,4-NQ) interference with polysulfide activation of SSP4. (**A**) SSP4 (5 µM) added to 10 µM of the polysulfide K_2S_n before addition of increasing concentrations of 1,4-NQ slightly increased, then decreased SSP4 fluorescence. (**B**) 1,4-NQ added to K_2S_n before SSP4 concentration-dependently decreased SSP4 fluorescence. (**C**) SSP4 fluorescence was unaffected when 1,4-NQ was added 60 min after incubating SSP4 with 10 µM K_2S_n. Mean +SEM, n=4 wells per treatment; **, *p<0.01*. ***, *p<0.001*.

Supplemental Figure S4. Effects of various naphthoquinones on polysulfide production (SSP4 fluorescence) from 300 µM H_2S in 21% O_2 or <1% O_2. Top panels (line graphs) illustrate the effects over time and are shown full scale, bottom panels (bar graphs) summarize responses at 100 min and scale is normalized to plumbagin. **A** and **B** are from same experiment. Mean +SEM, wells per treatment; **, *p<0.01*; ***, *p<0.001* compared to corresponding treatment in 21% O_2.

Supplemental Figure S5. Typical traces of the effects of SOD (0.1 µM) on oxygen consumption by 300 µM H_2S or 300 µM dithiothreitol (DTT) and various naphthoquinones. (**A**) SOD added to H_2S does not affect O_2 consumption. (**B-G**) Effects of SOD on O_2 consumption by H_2S and 10 µM of the NQs indicated in the figure. Buffer was equilibrated in room air (100% O_2) prior to the start of the experiment.

Supplemental Figure S6. Effects of reductants and ROS scavengers on oxygen consumption and H_2S oxidation by 10 µM NQs. (**A, B**) Typical oxygen consumption traces of various combinations of 1,4-NQ (10 µM), dithiothreitol (DTT, 300 µM), and SOD (0.1 µM) without (**A**) or with (**B**) 300 µM H_2S. SOD does not increase oxygen consumption without or with H_2S and DTT with 1,4-NQ consume more oxygen than 1,4-NQ with H_2S. (Note in **B**: H_2S+DTT+1,4-NQ+SOD shows duplicate experiments.) (**C**) Polysulfide production (SSP4 fluorescence) by 1 mM H_2S alone and by various combinations of 1 mM DTT, 1 mM 1,4-NQ and 0.1 µM SOD. DTT does not affect SSP4 fluorescence when added to H_2S or H_2S plus 1,4-NQ, SOD

significantly ($p<0.01$) decreases fluorescence when added to H_2S+1,4-NQ+DTT; mean +SEM, wells per treatment. (**D**) Effects of 300 μM ascorbic acid (AA) on oxygen consumption alone and in the presence of 10 μM 1.4 NQ and 0.1 μM SOD. Neither 1,4-NQ nor AA increase oxygen consumption, whereas together oxygen consumption is greatly increased. Subsequent addition of SOD does not affect oxygen consumption. (**E**) Effects of 300 μM H_2S on oxygen consumption by 300 μM AA, 10 μM 1,4-NQ and 0.1 μM SOD. Both 1,4-NQ and AA increase oxygen consumption when added to H_2S and consumption is further increased when 1,4-NQ and AA are added to H_2S. SOD does not affect consumption by H_2S plus 1,4-NQ and AA. (**F**) Effects of the free racical scavenger, tempol (Tem, μM) and the hydroxyl radical scavenger, mannitol (Man, 1 mM) on oxygen consumption by H_2S and 1,4-NQ. Tem and Man alone do not consume oxygen, Man does not affect oxygen consumption by H_2S and 1,4-NQ, whereas, Tem increases consumption by H_2S and 1,4-NQ. (**G-J**) Effects of SOD on O_2 consumption by 300 μM DTT and 10 μM of juglone, menadione, 2-MNQ and lawsone, respectively.

Supplemental Figure S7. Time-resolved absorption spectra (1.4 to 1576 sec) of H_2S reaction with 1,4-NQ; concentrations for each row indicated on the left. (**A**) H_2S only, (**B**) H_2S plus 1,4-NQ, (**C**) H_2S plus 1,4-NQ minus H_2S only at each time point (column **B** minus **A**), (**D**) deconvoluted spectra showing three consecutive components, a, b and c, (**E**) absorption peaks 208, 228 and 252 nm as a function of time.

Supplemental Figure S8. NQ reactions with H_2S do not increase DCF fluorescence. (**A**) 300 μM H_2S plus variable 1,4-NQ, (**B**) 30 μM 1,4-NQ plus variable H_2S, (**C**) 300 μM 1,4-NQ plus variable H_2S, (**D-I**) 300 μM H_2S plus variable juglone (Jug; **D**), plumbagin (Pbn; **E**), lawsone (Law; **F**), phylloquinone (Phyl; **G**), menaquinone (Mena; **H**) and 2-methoxy-1,4-naphthoquinone (2MNQ; **I**). DCF fluorescence was slightly decreased by 100 μM of all naphthoquinones; there were no consistent trends with variable H_2S and either 30 or 300 μM 1,4-NQ. Mean +SEM, n=4 wells per treatment; **, $p<0.01$; ***, $p<0.001$ compared to control; bar graphs summarize results at 90 min.

Supplemental Figure S9. DTPA (diethylenetriamine pentaacetic acid) does not affect NQ-catalyzed polysulfide production (SSP4 fluorescence). Samples were incubated with 300 μM H_2S and variable 1,4-NQ without (0 DTPA) or with 50 μM DTPA (+DTPA). Mean +SEM, n=4 wells per treatment; bar graphs summarize results at 90 min. There were no significant differences between 0 DTPA and + DTPA.

Supplemental Fig. S1

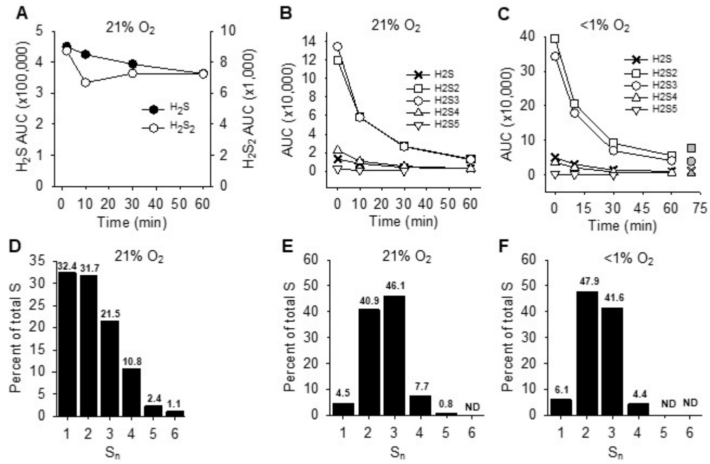

Supplemental Fig. S2

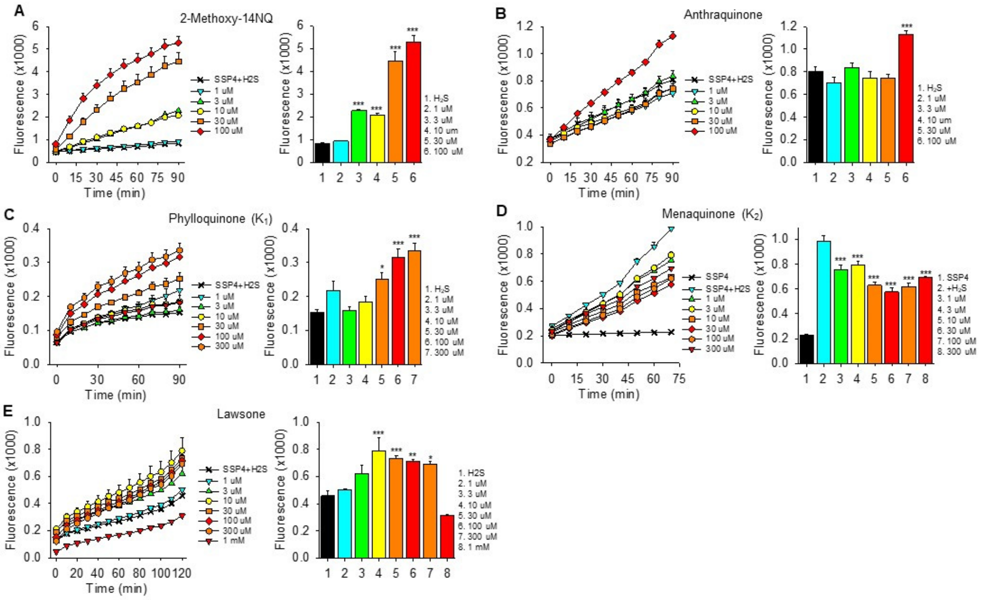

Supplemental Fig. S3

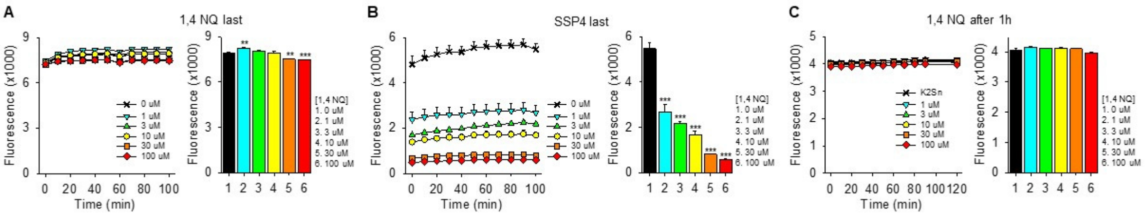

Supplemental figure S4

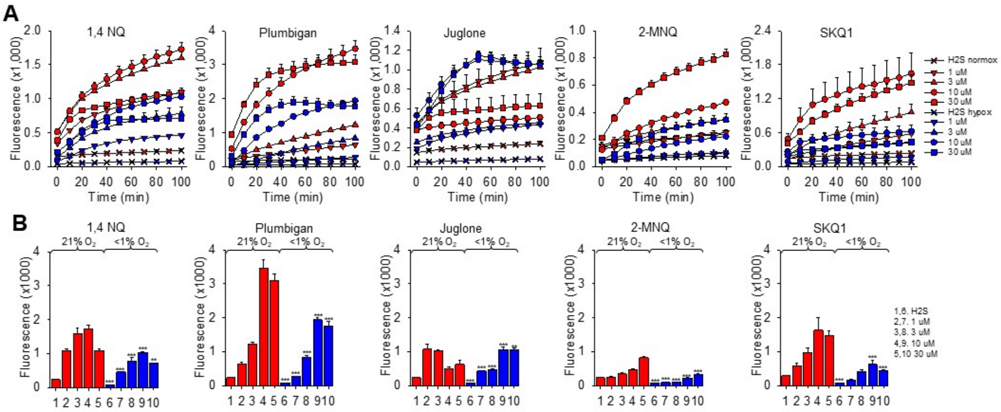

Supplemental Fig. S5

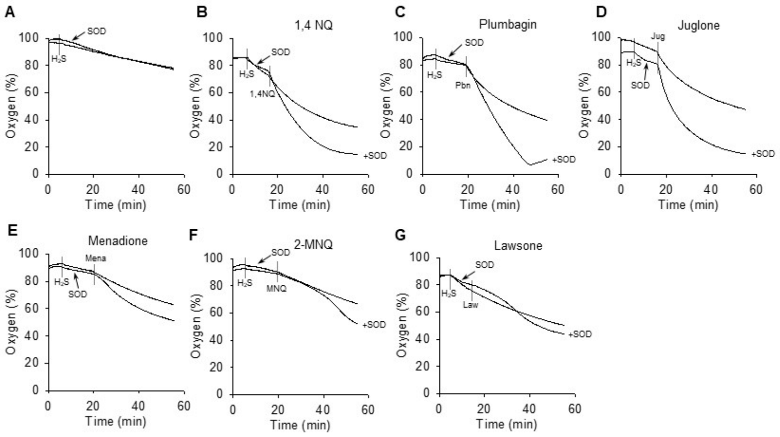

Supplemental Fig. S6

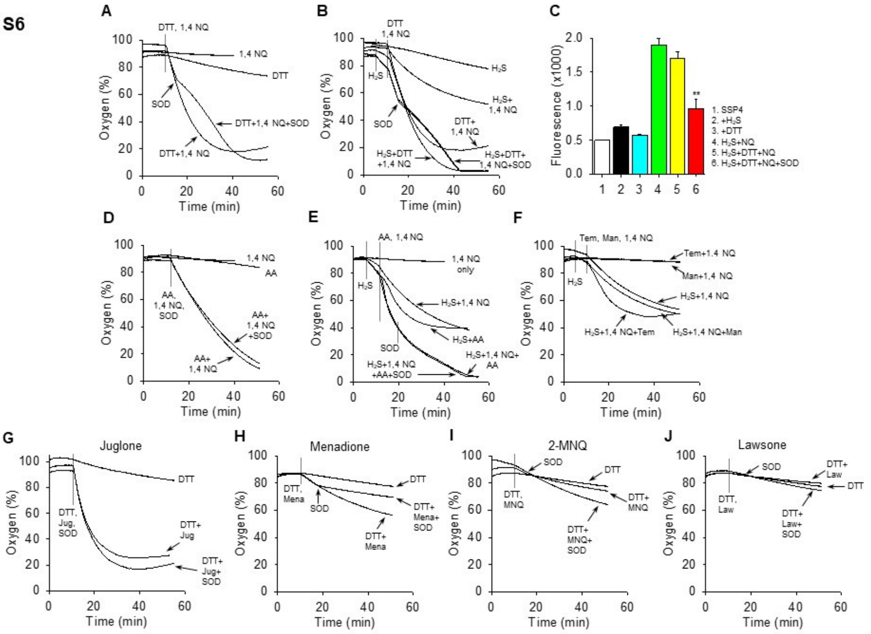

Supplemental Fig. S7

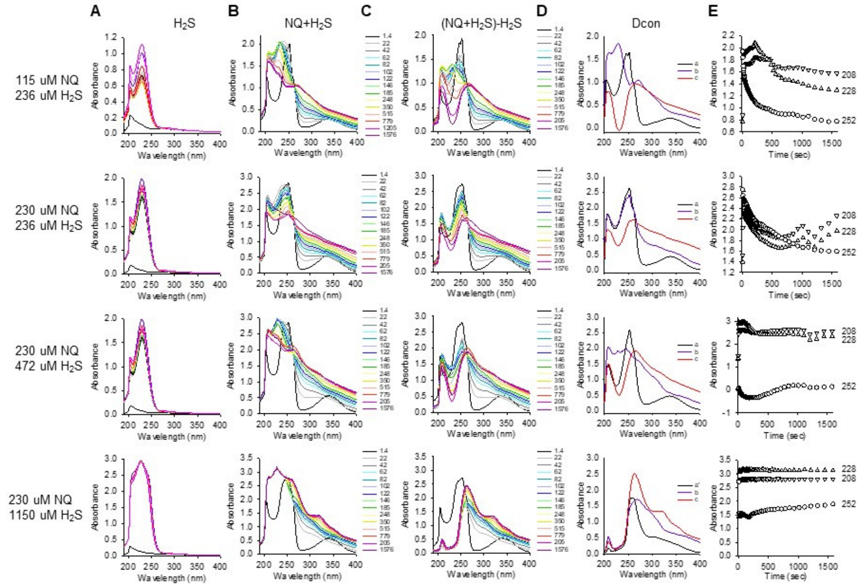

Supplemental Fig. S8

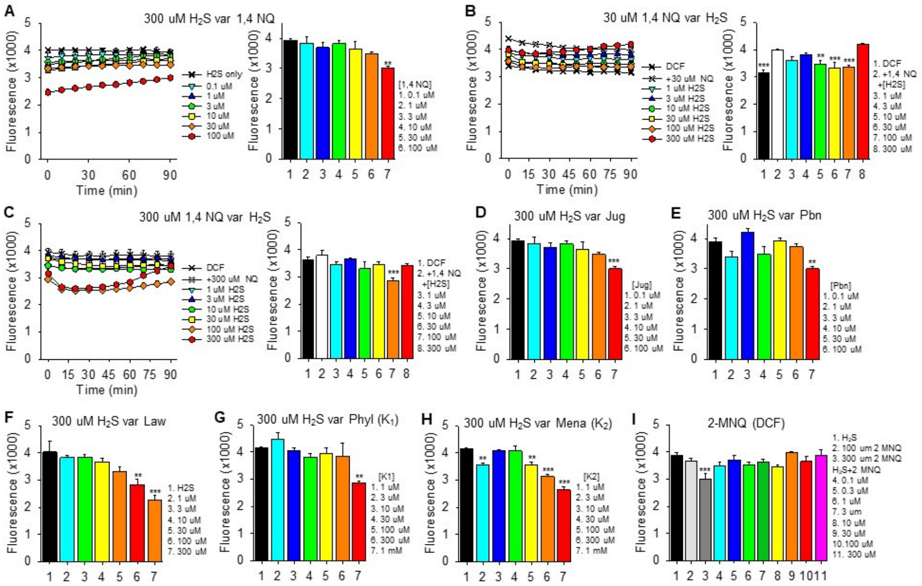

Supplemental Fig. S9

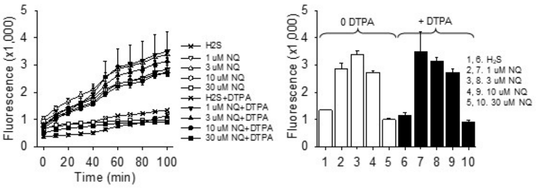

Supplement: Supplementary file 1 [file ijms-23-13293-s001.zip › ijms-1880655-supplementary.pdf]
